# Single-cell analysis reveals landscape of endometrial cancer response to estrogen and identification of early diagnostic markers

**DOI:** 10.1371/journal.pone.0301128

**Published:** 2024-03-22

**Authors:** Chunli Dong, Liyan Zhao, Xiongtao Liu, Ling Dang, Xin Zhang

**Affiliations:** 1 Department of Anesthesiology and Operation, The Second Affiliated Hospital of Xi’an Jiaotong University, Xi’an, China; 2 Department of Obstetrics and Gynecology, The Second Affiliated Hospital of Xi’an Jiaotong University, Xi’an, China; University of Salerno, ITALY

## Abstract

**Background:**

The development of endometrial cancer (EC) is closely related to the abnormal activation of the estrogen signaling pathway. Effective diagnostic markers are important for the early detection and treatment of EC.

**Method:**

We downloaded single-cell RNA sequencing (scRNA-seq) and spatial transcriptome (ST) data of EC from public databases. Enrichment scores were calculated for EC cell subpopulations using the “AddModuleScore” function and the AUCell package, respectively. Six predictive models were constructed, including logistic regression (LR), Gaussian naive Bayes (GaussianNB), k-nearest neighbor (KNN), support vector machine (SVM), extreme gradient boosting (XGB), and neural network (NK). Subsequently, receiver-operating characteristics with areas under the curves (AUCs) were used to assess the robustness of the predictive model.

**Result:**

We classified EC cell coaggregation into six cell clusters, of which the epithelial, fibroblast and endothelial cell clusters had higher estrogen signaling pathway activity. We founded the epithelial cell subtype *Epi cluster1*, the fibroblast cell subtype *Fib cluster3*, and the endothelial cell subtype *Endo cluster3* all showed early activation levels of estrogen response. Based on EC cell subtypes, estrogen-responsive early genes, and genes encoding Stage I and para-cancer differentially expressed proteins in EC patients, a total of 24 early diagnostic markers were identified. The AUCs values of all six classifiers were higher than 0.95, which indicates that the early diagnostic markers we screened have superior robustness across different classification algorithms.

**Conclusion:**

Our study elucidates the potential biological mechanism of EC response to estrogen at single-cell resolution, which provides a new direction for early diagnosis of EC.

## Introduction

Endometrial cancer (EC) is the most common malignant tumor that occurs in women, and it is one of the leading causes of death in women [1]. In 2018 alone, there were 89,929 EC deaths and 382,069 new cases [2, 3]. The precursor to endometrial adenocarcinoma has now been identified by pathologists as cellular heterogeneity in biopsy samples from EC [4]. In light of this, a comprehensive understanding of the tumorigenic mechanisms of EC will help improve the diagnosis and treatment of the disease.

Estrogen has been demonstrated to be able to go on to enhance the malignant biological behavior of EC through the mediation of the estrogen receptor (ER) [5]. To date, ER has been shown to have a role in promoting malignant characteristics of tumors in a variety of cancers [6, 7]. In addition, the development of EC is also thought to be associated with localized low-dose estrogen stimulation [8]. During the development of EC with estrogen-dependent properties, the endometrium is exposed to estrogen in the absence of progesterone protection and triggers proliferation, which in turn contributes to the development of a normal endometrium to atypical endometrial hyperplasia, and finally to endometrioid endometrial cancer (EEC). Based on histopathologic features, EC has been classified into two types: estrogen-dependent (type I) and estrogen-independent (type II). Of these, type I EC, which accounts for about 85% of endometrial cancer cases, typically expresses high levels of ER and is thought to be estrogen driven [9]. Although patients with type II EC have a poorer prognosis, the mortality rate of estrogen-driven type I EC is increased by its high prevalence [10]. Hu et al. demonstrated that ER can promote or inhibit cell proliferation by regulating the expression of p21 and CyclinD1 in EC cells [11]. Qi et al. showed that estrogen stimulation promoted the activation of the Ras-Raf-MEK-ERK and PI3K/Akt signaling pathways and increased expression of human MOF, which in turn promoted cancer cell proliferation [12]. However, due to the lack of early symptoms of EC occurrence, this can lead to the majority of patients being in advanced stages of EC by the time they are diagnosed. Thus, diagnostic markers with high sensitivity and specificity are urgently needed for the early diagnosis of EC.

In the present study, we assessed the heterogeneity of different EC cell subpopulations in response to estrogen based on single-cell transcriptional profiling of EC, including enrichment analysis to probe the activation status of estrogen-related signaling pathways in the cells, as well as the distribution of expression and proliferation of key estrogen-related genes. In addition, we screened for differentially expressed genes (DEGs) in key EC cell subpopulations to identify estrogen-associated markers for early diagnosis of EC. Finally, multiple machine learning methods were used to assess the robustness of the candidate markers. Our study provides valuable insights into the early diagnosis and treatment of EC.

## Methods

### Acquisition of data

Based on the Gene Expression Omnibus (GEO, https://www.ncbi.nlm.nih.gov/geo/) database, single-cell RNA-seq (scRNA-seq) data of 5 endometrioid adenocarcinoma (EAC) patients (including GSM5276933, GSM5276934, GSM5276935, GSM5276936, and GSM5276937) and spatial transcriptome (ST) data of 1 EC patient (GSM6177623 in GSE203612) were downloaded separately.

In addition, we downloaded data on uterine corpus endometrial carcinoma (UCEC) based on the Cancer Genome Atlas (TCGA) website (https://portal.gdc.cancer.gov/), which contained a total of 589 samples. To screen for biomarkers for early diagnosis, we screened only Stage I and para-cancerous tissue samples for further analysis.

### Processing of single-cell RNA sequencing data

First, we analyze the scRNA-seq data based on the "Read10X" function in the Seurat package. We retained cells that expressed gene numbers ranging from 300 to 7,500 and had unique molecular identifiers (UMIs) greater than 25% of the mitochondrial genome. Subsequently, we removed mitochondrial, ribosomal, and hemoglobin genes from this dataset and ultimately retained 26,145 cells for further analysis.

Use the "SCTransform" function to normalize the data. After performing principal component analysis (PCA) on genes with high variability, we used the harmony package [13] to remove batch effects between samples (max.iter.harmony = 20, lambda = 0.5). Next, the top 40 principal components (PCs) were used for uniform manifold approximation and projection (UMAP) dimension reduction and the cells were clustered using the “FindNeighbors” and “FindClusters” functions (resolution = 0.1). We annotated cell clusters according to the cell types available in CellMarker 2.0 (http://117.50.127.228/CellMarker/). Among them, epithelial cells, fibroblasts and endothelial cells were downscaled by UMAP using only the top 5 PCs. Finally, we used the "FindAllMarkers" function of the Seurat package to distinguish differentially expressed gene (DEGs) in different cell clusters (only.pos = T, min.pct = 0.25, logfc.threshold = 0.25).

### Expression of key genes of interest in the spatial transcriptome

We did this by using the Seurt package (version 4.2) in order to analyze spatial transcriptome data. The "SpatialFeaurePlot" function is used to view the distribution of a specific gene of interest on EC tissue.

### Functional enrichment analysis

Gene Oncology (GO) enrichment analysis to characterize biological processes (BP) that do not make sense of cellular subpopulations. Based on our screening of the genes of interest, the "enrichGO" function in the clusterprofiler package [14] was used to perform functional enrichment analysis. The relevant parameters were set as follows: keyType = "SYMBOL", pvalueCutoff = 0.05, qvalueCutoff = 0.1, and ont = "BP".

### Calculation of enrichment scores of cell subpopulations for estrogen-related gene sets

First, we downloaded the hallmark gene set from the Molecular Signatures Database (MsigDB, https://www.gseamsigdb.org/gsea/msigdb/download_file.jsp?filePath=/msigdb/release/2023.1.Hs/h.all.v2023.1.Hs.symbols.gmt) and obtained the gene sets for Hallmark estrogen response early and Hallmark estrogen response late signaling pathways, respectively, from this gene set. Subsequently, the gene set of the estrogen signaling pathway was obtained by Kyoto Encyclopedia of Genes and Genomes (KEGG) and the expression matrix of cell subpopulations was extracted. Finally, the gene set scores within each sample were calculated using the AUCell R package [15] and the "AddModuleScore" function, respectively.

### Construction of machine learning models

We based our samples on the TCGA-UCEC dataset and extracted the expression matrix and features of the hallmark estrogen response early gene. We divide the eligible data into training and validation cohorts in the ratio of 7:3. Subsequently, six methods including logistic regression (LR), Gaussian naive Bayes (GaussianNB), k-nearest neighbor (KNN), support vector machine (SVM), eXtreme gradient boosting (XGB), and neural network (NK) were used to construct the classifier. Among them, the LR model is commonly used to study the effect of feature variables on target variables to solve the problem of binary classification [16]. GaussianNB is a probabilistic model with high specificity and sensitivity accuracy [17]. The KNN model is a supervised machine learning method that can be used for classification and regression tasks [18]. SVM, on the other hand, is a kernel-based algorithm that is able to transform a feature space with multidimensional attributes into two classes [19]. The XGB classifier is using gradient boosting and its ability to improve the performance of the model based on the difference between the true and predicted values [20]. In addition, the NK is one of the machine learning methods that can be used for prediction and classification, which is in a sense modeling the impulse propagation mechanism in the nervous system [21]. Finally, in order to assess the performance of these six predictive models, we evaluated the diagnostic efficacy of each classifier by building receiver‐operating characteristic (ROC) curves, as well as using the areas under the ROC curves (AUCs) in order to assess the diagnostic efficacy of each classifier.

### Statistical analysis

In this study, we used R software (version 4.3.1) and Python language (version 3.11.4) for statistical analysis. The Wilcoxon test was used to compare the differences in continuous variables between the two groups. Specifically, *p* <0.05 was considered statistically significant.

## Results

### Classification of single-cell subpopulations of EC and identification of marker genes

**Fig 1:**
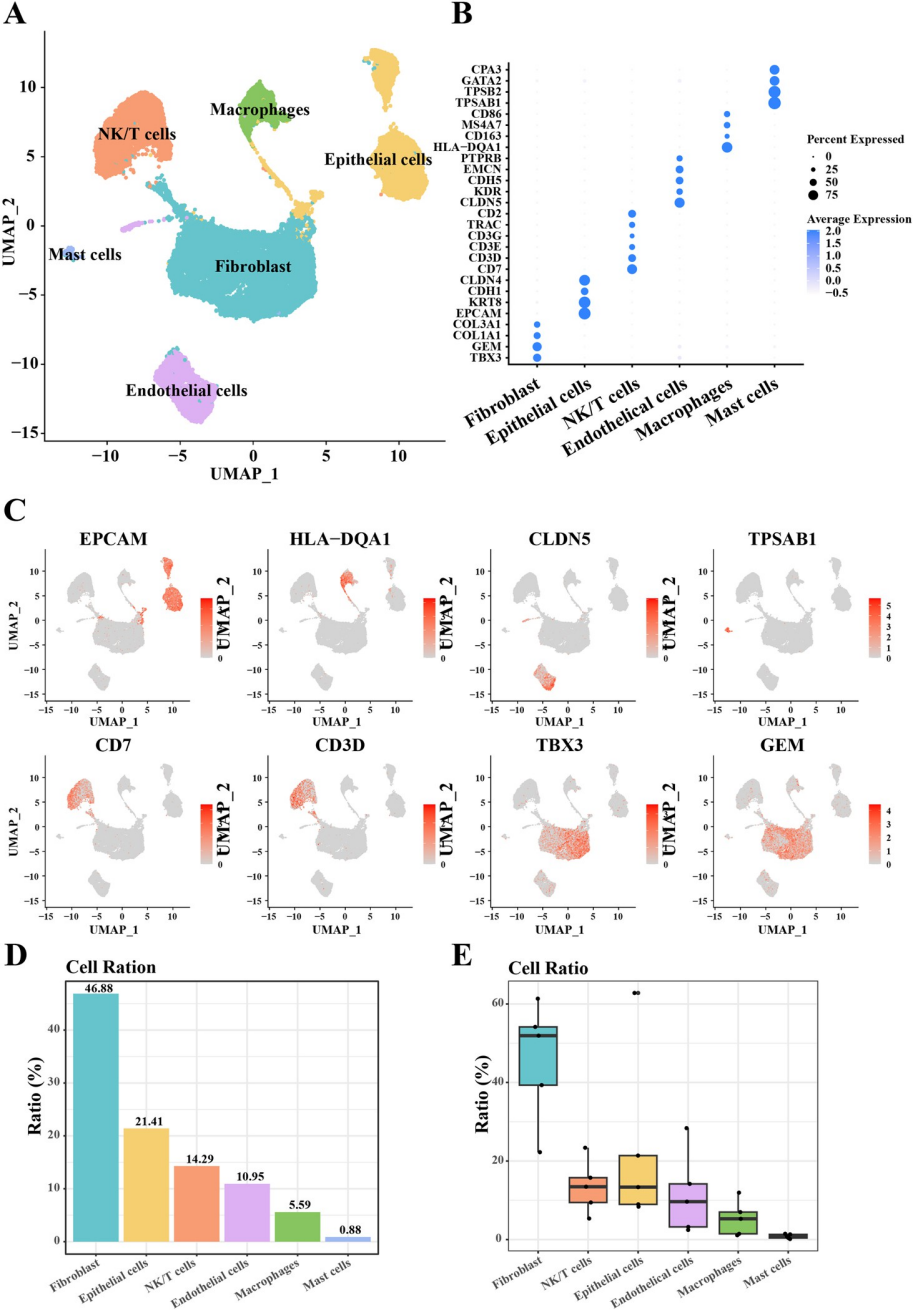
Classification of single-cell subpopulations of EC and identification of marker genes. (A) UMAP plot of annotated cell types in EC; (B) Bubble plot showing gene expression in six cell clusters; (C) Violin plot showing marker gene expression in EC cell subpopulations; (D) Percentage of each cell subpopulation within all samples (5 patients with EC); (E) Demonstration of the percentage of each subpopulation in EC cell samples.

First, we obtained six major cell clusters including fibroblasts, epithelial cells, NK/T cells, endothelial cells, macrophages, and mast cells based on the Seurat package and after normalization, downscaling, and clustering of scRNA-seq data (**Fig 1A**). In addition, **Fig 1B and 1C** show the expression levels of representative marker genes for each cell type. We found that genes such as EPCAM, KRT8, CDH1, and CLDN4 were highly expressed in epithelial cells. CLDN5, KDR, CDH5, EMCN, and PTPRB genes are highly expressed in endothelial cells. Mast cells highly express TPSAB1, TPSB2, GATA2, CPA3 genes. NK/T cells highly expressed CD7, CD3D, CD3E, CD3G, TRAC, CD2. In addition, TBX3, GEM, COL1A1, and COL3A1 genes were significantly highly expressed in fibroblasts. Subsequently, we based the percentage of each cell cluster in all samples and cells of EC (**Fig 1D and 1E**). We observed that among these six cell types, the percentage of fibroblast clusters was the highest (46.88%), followed by epithelial cells (21.42%) and NK/T cells (14.29%).

#### Heterogeneity of cell subpopulations in EC in response to estrogen

**Fig 2:**
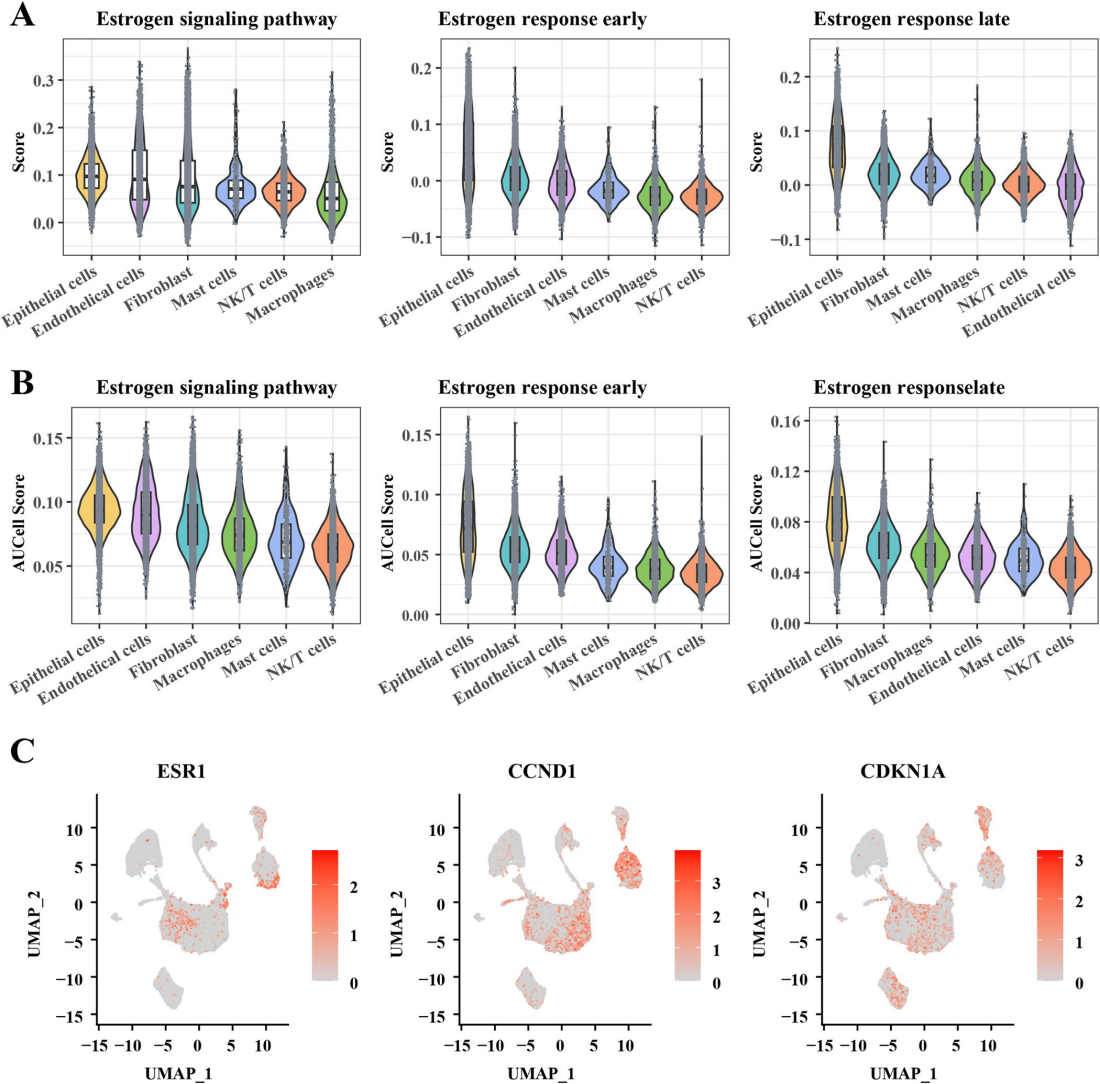
Relationship of different cell clusters of EC to estrogen signaling pathways. (A-B) Differences in enrichment scores for estrogen signaling pathways in different cell subpopulations based on the AddModuleScore function (A) and AUCell package (B); (C) Distribution of three key estrogenic pathways and cell proliferation-promoting genes (including ESR1, CCND1, and CDKN1A) in the EC.

To explore the activation status of the estrogen signaling pathway in cells, we based our enrichment scores on estrogen-related genes in HALLMARK and KEGG with the “AddModuleScore” function and the AUCell package, respectively. As shown in **Fig 2A**, we found that compared to other cell clusters, fibroblasts, epithelial cells and endothelial cells had higher enrichment scores in the estrogen signaling pathway. Meanwhile, we observed significant enrichment of epithelial, fibroblast and endothelial cell clusters in the early estrogen response. In addition, enrichment scores in the late estrogen-responsive pathway showed enhanced mast cell and macrophage responses to estrogen. In particular, we find that the enrichment results based on the AUCell score (**Fig 2B**) are the same as those based on the “AddModuleScore” function. These results confirm that different cellular subpopulations of the EC are heterogeneous in their activation status in the estrogen signaling pathway. Notably epithelial cells, endothelial cells and fibroblasts have a strong correlation with early estrogen response.

Estrogen Receptor 1 (ESR1) is a key member of the estrogen signaling pathway that encodes ER-α. Typically, ESR1 binds to estrogen in order to regulate biological processes such as cell proliferation, differentiation and apoptosis [22, 23]. Cyclin D1 (CCND1) is an important molecule in cell cycle regulation that promotes proliferation and apoptosis in human cancer cells [24, 25]. In addition, CDKN1A, as a gene capable of inhibiting cell cycle protein-dependent kinases, is considered to be an important tumor suppressor in the pathogenesis of cancer [26]. To explore the subpopulations of cells in the EC in promoting estrogen signaling pathway activation and cell proliferation, we visualized the expression of ESR1, CCND1 and CDKN1A in endothelial cells, fibroblasts and epithelial cells of the EC. As shown in **Fig 2C**, we observed that ESR1 was predominantly distributed in the epithelial cells of ECs, followed by fibroblasts. And by observing the difference in the distribution of CDKN1A and CCND1 on EC single-cell profiles, it was shown that there was great heterogeneity of epithelial, endothelial, and fibroblasts in the EC. Therefore, it is necessary to further explore the differences in the response of epithelial, endothelial, and fibroblast clusters to estrogen in the EC separately.

### Heterogeneity of epithelial cell subpopulations in EC in response to estrogen

**Fig 3:**
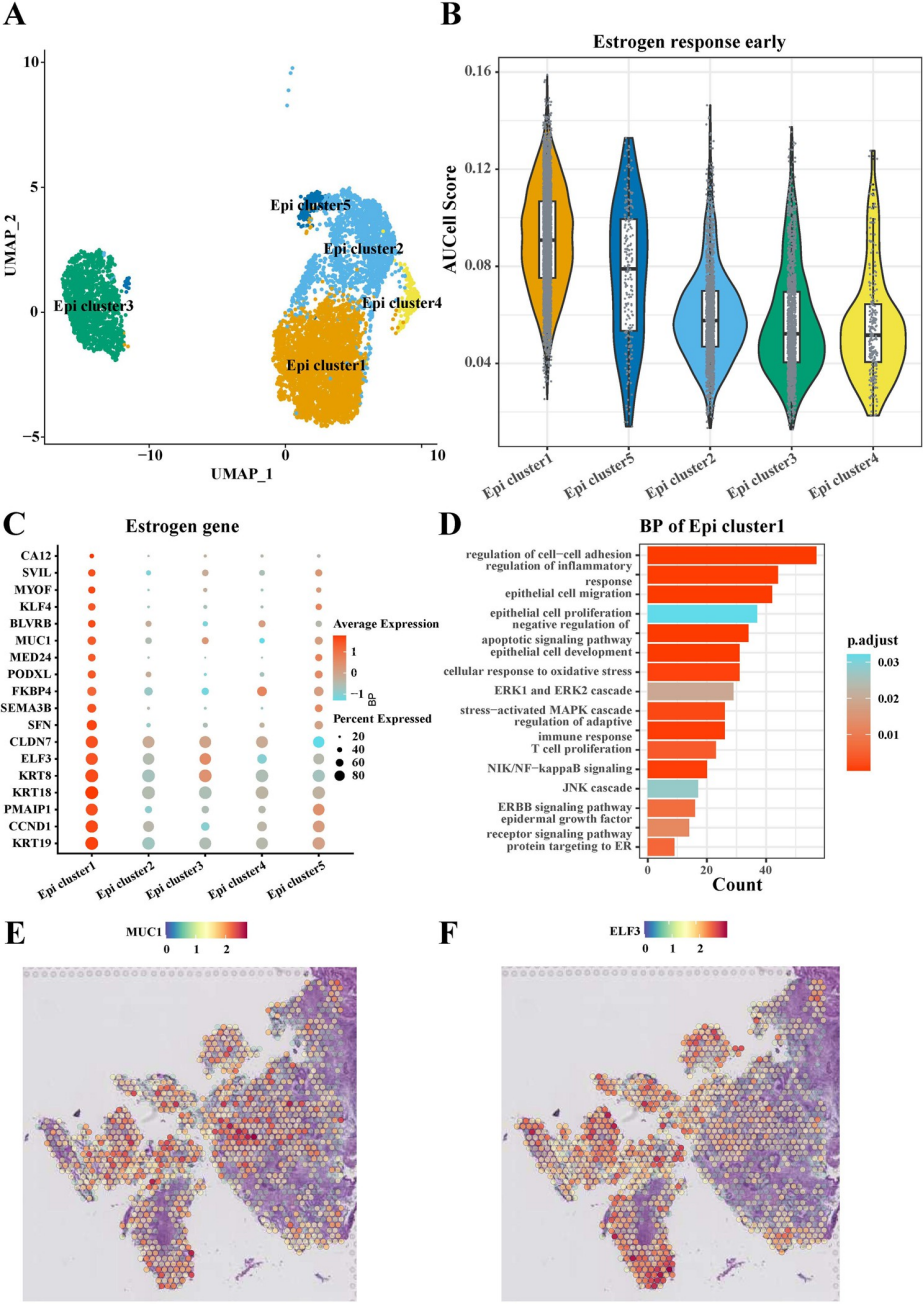
Response of epithelial cells to estrogen in the EC. (A) UMAP showed classification into 5 cell subpopulations based on 7,162 epithelial cells; (B) Comparison of the level of early activation of five epithelial cell subpopulations in response to estrogen; (C) Differential expression of estrogen-related gene levels in different epithelial cell subpopulations; (D) Demonstration of enrichment analysis of biological processes of genes in *Epi cluster1*; (E-F) Distribution of MUC1 (E) and ELF3 (F) genes in EC tissues.

To understand the relationship between the heterogeneity of EC epithelial cells and the activation of the estrogen signaling pathway, we obtained a total of 7,162 epithelial cells for the identification of epithelial cell subpopulations. We showed five epithelial cell subpopulations based on epithelial cell data and using UMAP plots for *Epi cluster1*, *Epi cluster2*, *Epi cluster3*, *Epi cluster4*, and *Epi cluster5* (**Fig 3A**). Specifically, AUCell enrichment scores showed that *Epi cluster1* had the highest level of early activation of the estrogen response (**Fig 3B**). In addition, most estrogen genes were also highly expressed in *Epi cluster1* compared to the other 4 subpopulations of epithelial cells, such as KRT19, CCND1, PMAIP1, KRT18, KRT8, ELF3, and MUC1 (**Fig 3C**). Next, we explored the biological processes of *Epi cluster1* by enrichment analysis and found that this subpopulation is mainly enriched in pathways such as epithelial cell proliferation and migration, and regulation of cell-cell adhesion (**Fig 3D**). MUC1 and ELF3 as estrogen-associated genes that are highly expressed in *Epi cluster1*, they are also known to influence cancer cell proliferation, migration and invasion. By visualizing the expression of MUC1 and ELF3 genes in their spatial locations in the EC, we found that these two genes were mainly enriched in the tumor region (**Fig 3E and 3F**).

### Heterogeneity of fibroblast subpopulations in EC in response to estrogen

**Fig 4:**
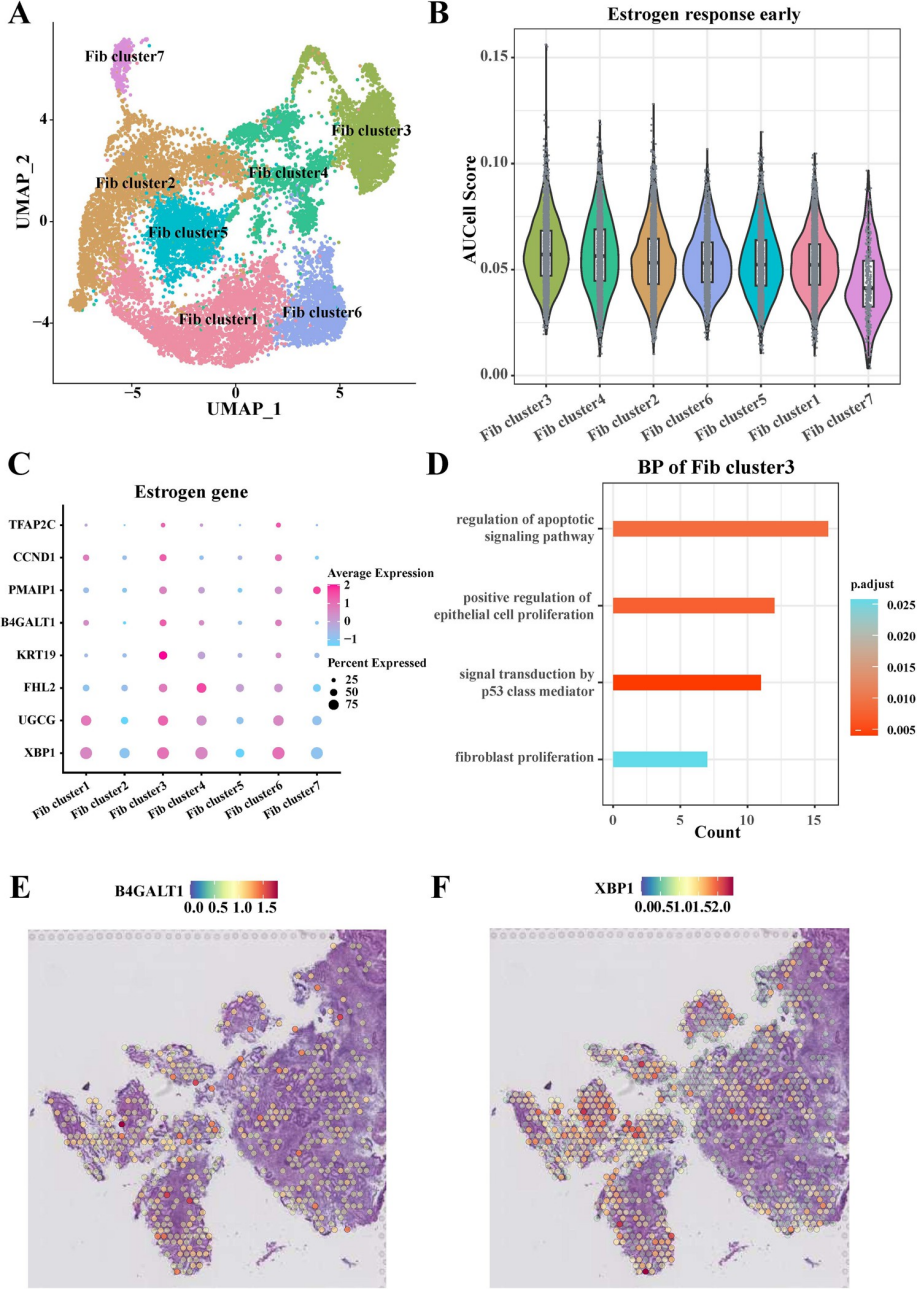
Response of fibroblasts to estrogen in the EC. (A) UMAP showed classification into 7 cell subpopulations based on 16,019 fibroblasts; (B) Comparison of the level of early activation of seven fibroblast subpopulations in response to estrogen; (C) Differential expression of estrogen-related gene levels in different fibroblast subpopulations; (D) Demonstration of enrichment analysis of biological processes of genes in *Fib cluster3*; (E-F) Distribution of B4GALT1 (E) and XBP1 (F) genes in EC tissues.

Totally, 16,019 fibroblast cells were detected and re-clustered into 7 clusters, including *Fib cluster1*, *Fib cluster2*, *Fib cluster3*, *Fib cluster4*, *Fib cluster5*, *Fib cluster6*, and *Fib cluster7* (**Fig 4A**). Among them, the AUCell enrichment score indicated that *Fib cluster3* had a higher level of early activation of estrogen response (**Fig 4B**) and that estrogen-related genes were expressed at higher levels in this subgroup (**Fig 4C**). In addition, the results of GO enrichment analysis indicated that the genes in F*ib cluster3* were mainly associated with the regulation of cell proliferation and autophagy signaling pathways (**Fig 4D**). We visualized the distribution of two estrogen genes, B4GALT1 and XBP1, which are highly expressed in *Fib cluster3*. The results showed that the expression of B4GALT1 and XBP1 was mainly enriched in the tumor region of EC (**Fig 4E and 4F**).

### Heterogeneity of endothelial cell subpopulations in EC in response to estrogen

**Fig 5:**
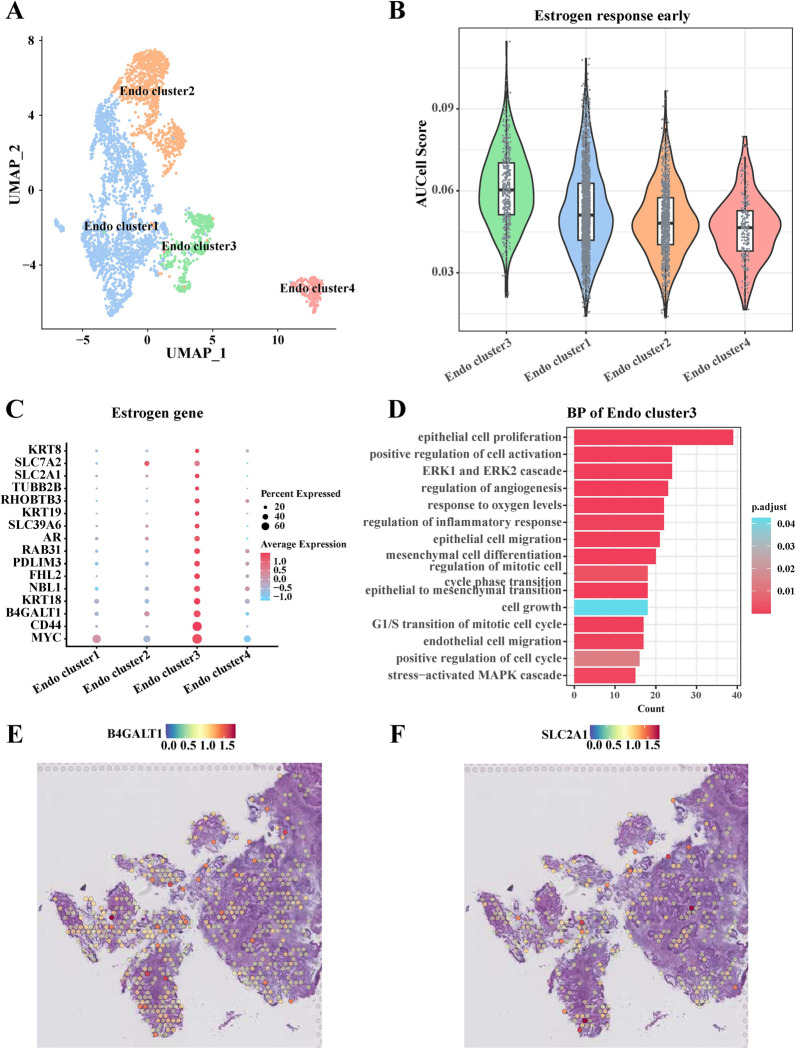
Response of endothelial cells to estrogen in the EC. (A) UMAP showed classification into 4 cell subpopulations based on 3,740 endothelial cells; (B) Comparison of the level of early activation of four endothelial cell subpopulations in response to estrogen; (C) Differential expression of estrogen-related gene levels in different endothelial cell subpopulations; (D) Demonstration of enrichment analysis of biological processes of genes in *Endo cluster3*; (E-F) Distribution of B4GALT1 (E) and SLC2A1 (F) genes in EC tissues.

We further clustered the 3,740 endothelial cells into four endothelial cell subpopulations, including *Endo cluster1*, *Endo cluster2*, *Endo cluster3*, and *Endo cluster4* (**Fig 5A**). AUCell enrichment scores indicate that *Endo cluster3* has the highest AUCell score (**Fig 5B**) and most estrogen genes are significantly highly expressed in this subgroup (**Fig 5C**). This suggests a higher level of early activation of *Endo cluster3* in response to estrogen. Furthermore, GO analysis also showed that *Endo cluster3* was mainly enriched in the pathways of cell proliferation, migration, positive regulation of cell activation, and epithelial-to-mesenchymal transition (**Fig 5D**). SLC2A1, a member of the SLC transporter protein family, is primarily involved in encoding glucose transporter proteins at the cell membrane and cell surface [27]. Importantly, SLC2A1 has been reported to promote cancer development by regulating cell proliferation [28]. To this end, we observed that the expression of both SLC2A1 and B4GALT1 was enriched in tumor tissue regions in the EC (**Fig 5E and 5F**).

### Identification of early diagnostic markers associated with estrogen response in EC

**Fig 6:**
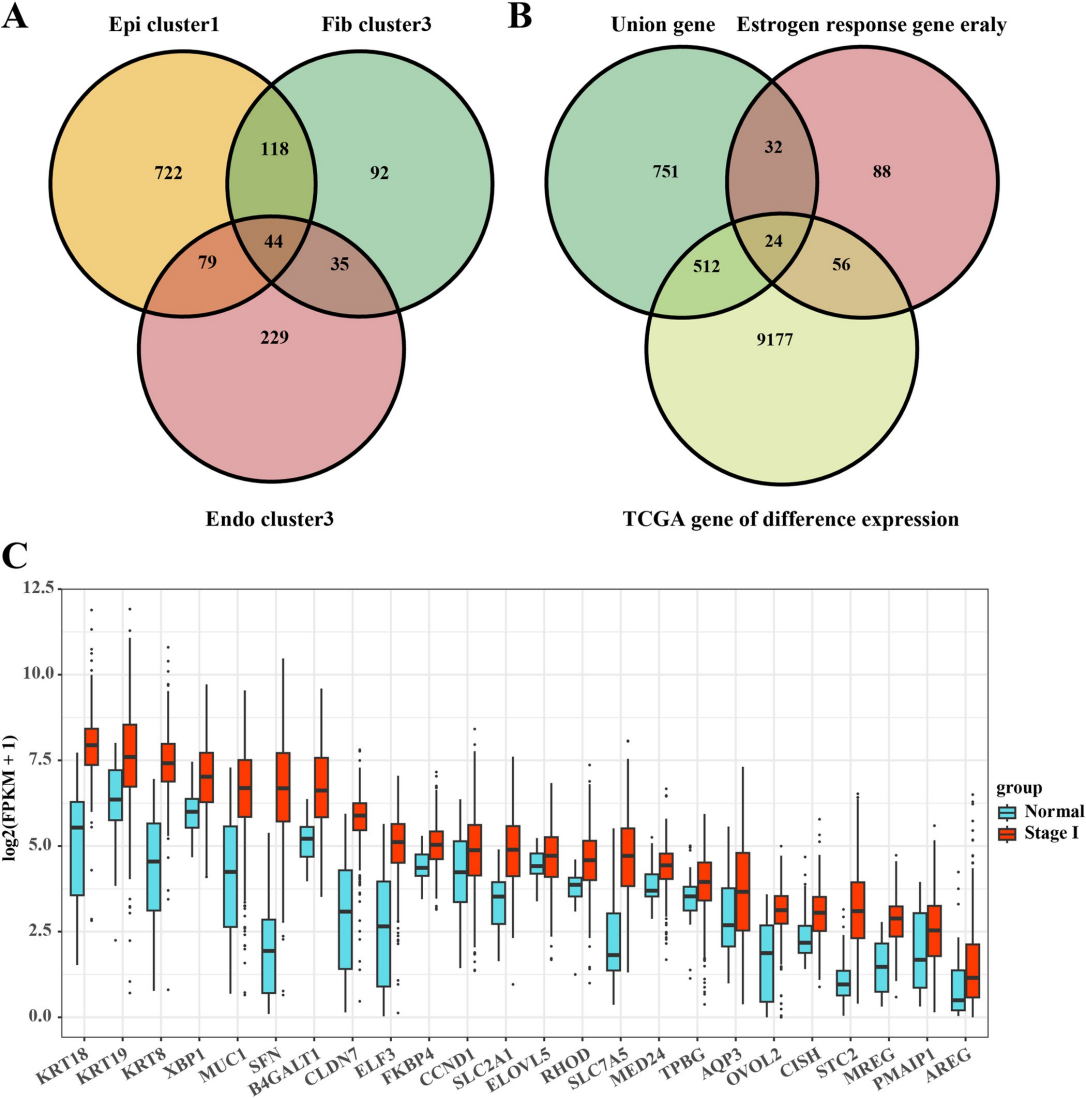
Identification of biomarkers of early response to estrogen in EC. (A) Screening of DEGs between cell subpopulations (including *Epi cluster1*, *Fib cluster3*, and *Endo cluster3*); (B) Screening of cellular subpopulations for DEGs as well as Stage I and para-cancer tissue genes in EC to identify diagnostic markers; (C) Expression levels of 24 early diagnostic markers of estrogen response in EC and its para-cancer tissues.

Based on the clustering of epithelial cells, fibroblasts and endothelial cells and the identification of marker genes, we next screened DEGs in *Epi cluster1*, *Fib cluster3*, and *Endo cluster3* (**Fig 6A**). Subsequently, we screened for early diagnostic markers of response to estrogen based again on DEGs among cellular subpopulations, early genes for estrogen response, and genes encoding differentially expressed proteins in Stage I and para-cancer in the TCGA-UCEC dataset. As shown in **Fig 6B**, we identified a total of 24 early diagnostic marker candidates responsive to estrogen in the EC, including KRT18, KRT19, KRT8, XBP1, MUCI, SFN, B4GALT1, CLDN7, ELF3, FKBP4, CCND1, SLC2A1, ELOVL5, RHOD, SLC7A5, MED24, TPBG, AQP3, OVOL2, CISH, STC2, MREG, PMAIPP1, and AREG. Furthermore, based on the Wilcoxon test and relative to normal tissue samples, we found that all of these 24 early diagnostic marker candidates were significantly highly expressed in tumor tissue (**Fig 6C**). These results further confirm that EC cells may signal through the estrogen early signaling pathway in order to promote cancer development.

### Construction of a classifier for EC early diagnostic markers

**Fig 7:**
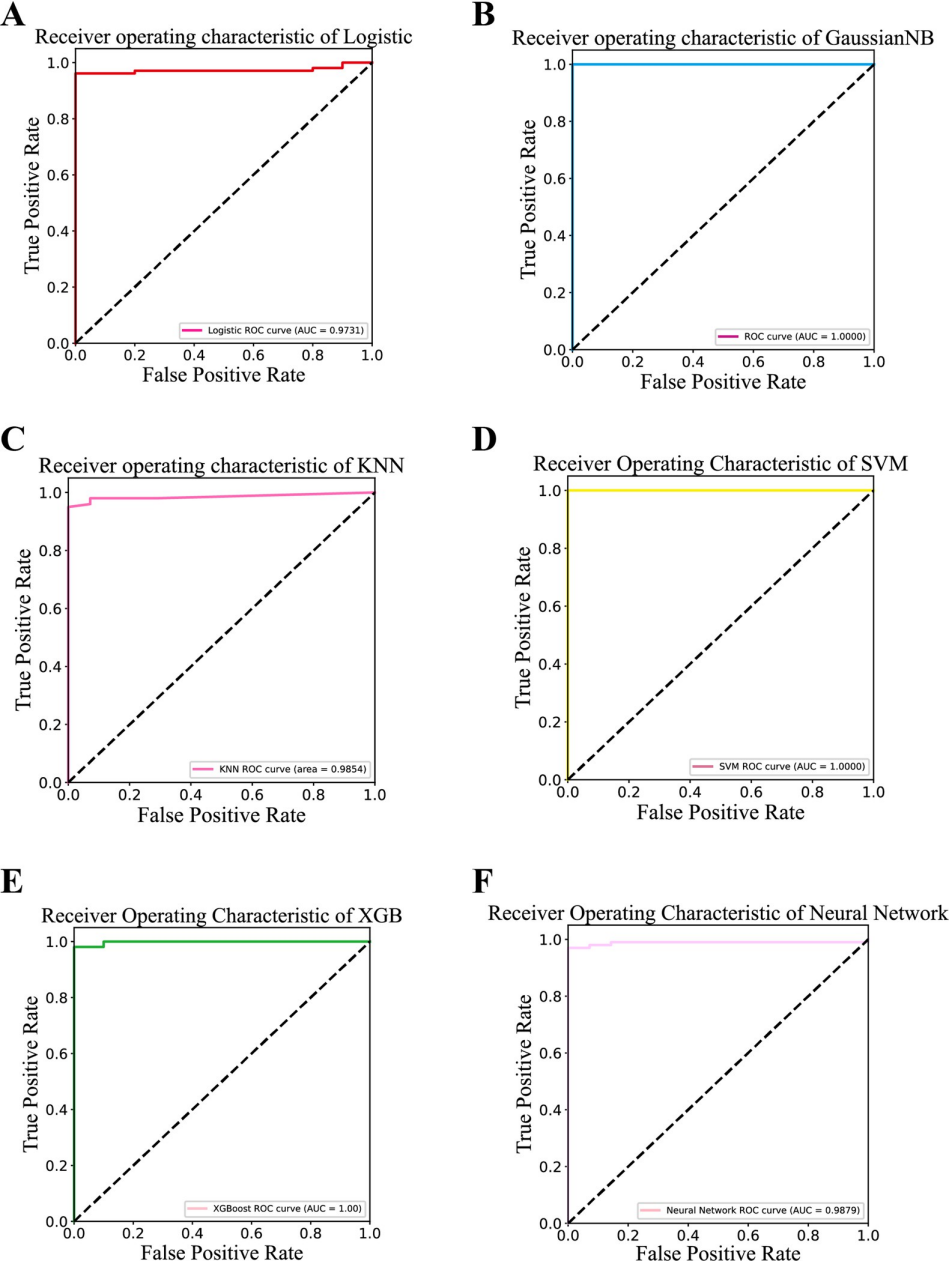
Predictive modeling of early diagnostic markers of estrogen response in EC. (A-F) Receiver operating characteristic of logistic regression (LR), Gaussian naive Bayes (GaussianNB), k-nearest neighbor (KNN), support vector machine (SVM), eXtreme gradient boosting (XGB), and neural network (NK).

To validate whether the 24 marker candidates can be applied to the early diagnosis of EC, we constructed diagnostic classifiers using six machine learning methods. The ROC curves for LR, KNN, and NK indicated AUC values of 0.9731, 0.9854, and 0.9879, respectively. Notably, the AUC values of GaussianNB, SVM and XGB are 1, respectively, which indicates that our screened candidate markers have excellent robustness in different classification algorithms (**Fig 7**).

## Discussion

In this study, we comprehensively analyzed single-cell data of EC and demonstrated the transcriptional and estrogenic regulatory landscape of EC, revealing the robust heterogeneity of EC cells.

In our study, EC cells were categorized into six clusters. Unlike previous studies, Yu et al. classified monocytes into 9 clusters including epithelial cells, T cells, fibroblasts, macrophages, NK cells, endothelial cells, B cells, Monocytes, and dendritic cells based on 4 EC and 2 normal endometrium samples. In addition, by analyzing the percentage of each type of cell, they found that epithelial cells (44.5%) had the highest percentage, followed by T cells (23.8%) and fibroblasts (19.1) [29]. However, in our study, fibroblast clusters (46.88%) accounted for the highest percentage, followed by epithelial cells (21.41%), NK/T cells (14.29%), and endothelial cells (10.95%). Possible reasons for the differences are differences in data sources and differences in the potential batch effects that the data have.

EC is a hormone-dependent disease in which estrogen plays an important role in the pathogenesis of EC due to its ability to induce histone acetylation [10, 30]. Lee et al. suggested that recurrent amplification boundaries and rearrangement hotspots occurring in breast cancer cells are associated with estrogen receptor binding. They found that estrogen treatment induces DNA double-strand breaks in the target region of the estrogen receptor, and that the breaks are subsequently repaired by translocation, revealing a mechanistic origin of estrogen in cancer [31]. In EC, Qi et al. found that estrogen and ER enhanced human MOF expression to promote cancer cell proliferation and inhibited apoptosis by activating the PI3K/Akt and Ras-Raf-MEK-ERK signaling pathways. However, the mechanism of estrogen-related signaling in EC is still blank. Thus, for the first time, we screened the EC cell clusters with the highest activation levels, including epithelial, fibroblast, and endothelial cell clusters, based on the estrogen-related signaling pathway enrichment score. Unlike the study of Regner et al. [32], we further clustered the three cell clusters into subtypes based on estrogen genes.

MUC1 is the most recognizable transmembrane protein in the mucin family and exerts a signaling function in cancer cells [33]. Specifically, MUC1 has been reported to be highly expressed in various epithelial adenocarcinomas, including ovarian, breast, lung and liver cancers [33–35]. Zhao et al. found that silencing MUC1 expression inhibited migration and invasion of pancreatic cancer cells [36]. In addition, MUC1 was able to enhance the invasiveness of cancer cells by inducing the transformation of the epithelial mesenchymal (EMT) [37]. ELF3, a transcription factor, is epithelial-specific and has been reported to be able to participate in cancer cell proliferation and migration, the EMT pathway, and epithelial tumor invasion [38–40]. Seo et al. found that ELF3 can promote the development of epithelial ovarian cancer by mediating the secretion of angiogenic factors [38]. In our study, we found that both MUC1 and ELF3 were highly expressed in the epithelial cell subtype *Epi cluster1* and densely distributed in the tumor region of EC. Also, the genes in *Epi cluster1* were enriched in the pathways of cancer cell proliferation and invasion. This suggests that EC epithelial cells are heterogeneous in their response to estrogen and suggests that MUC1 and ELF3 can serve as markers for targeting EC epithelial cells.

In addition, our study also confirmed that fibroblast and endothelial cell subtypes in the EC are also highly heterogeneous in their response to estrogen. Notably, B4GALT1, XBP1 and SLC2A1 were highly expressed in fibroblast subtypes and endothelial cell subtypes, all of which were enriched in the tumor region, and both *Fib cluster3* and *Endo cluster3* were associated with pathways such as cell proliferation and invasion. Cui et al. demonstrated that B4GALT1 is a key analysis in the early development of lung adenocarcinoma, and that it regulates the N-linked glycosylation of PD-L1 protein to induce immune escape from cancer [41]. XBP1 acts as a transcription factor which is able to participate in the stress response of the built-in web. It has been reported that the genetic characterization of XBP1 is strongly and positively correlated with the positive status of ER [42]. Wang et al. showed that knocking down XBP1 in mouse granulosa cells promotes apoptosis and inhibits the cell cycle [43]. In addition, XBP1 deficiency inhibits the proliferation of breast cancer cells [44]. SLC2A1 is a member of the SLC transporter protein family and is able to participate in a variety of cell death modalities. Wang and his colleagues comprehensively summarized the prognostic role of SLC2A1 in pan-cancer and found that SLC2A1 is able to be overexpressed in a wide range of tumors and is associated with a poor prognosis in patients [27]. Moreover, SLC2A1 has been shown to be a key gene in tumor glucose metabolism, which promotes glycolysis in cancer cells and thus affects the growth and metastasis of cancer cells [45, 46]. In addition to MUC1, ELF3, B4GALT1, XBP1 and SLC2A1, 19 other genes (including KRT18, KRT19, KRT8, SFN, CLDN7, FKBP4, CCND1, ELOVL5, RHOD, SLC7A5, MED24, TPBG, AQP3, OVOL2, CISH, STC2, MREG, PMAIP1, and AREG) were screened by us to serve as early diagnostic markers of EC response to estrogen. To date, KRT18 [47], KRT19 [48], KRT8 [49], SFN [50], CLDN7 [51], FKBP4 [52], CCND1 [53], ELOVL5 [54], RHOD [55], SLC7A5 [56], MED24 [57], TPBG [58], AQP3 [59], OVOL2 [60], CISH [61], STC2 [62], PMAIP1 [63], and AREG [64] have been reported to be highly expressed in a variety of cancers and associated with the promotion of cancer cell growth, proliferation, migration and invasion. Only MREG was found to be down-regulated in expression in thyroid cancer tissues, and knockdown of MREG promoted cancer cell proliferation and invasion [65]. These studies have amply demonstrated that key genes screened based on estrogen signaling can serve as promising biomarkers for early diagnosis and treatment of EC.

However, there are some limitations to this study. In our study, the sample size was limited to the extent that there was a lack of sufficient EC reference data to fully validate the role of estrogen-related genes in EC patients. Moreover, our findings are derived from an analysis of public databases, and more clinical cases are needed to validate our conclusions. Importantly, further complementary validation cohorts to validate the sensitivity and specificity of classifiers constructed for EC early diagnostic markers are also necessary.

## Conclusion

Overall, our study reveals the heterogeneity of cellular responses to estrogen in the EC. Further clustering of the major cell clusters of EC, including epithelial, fibroblast and endothelial cell clusters, was performed to demonstrate that EC is closely associated with aberrant activation of the estrogen signaling pathway. Importantly, we identified the cellular subtypes most associated with early estrogen response as well as screened 24 highly sensitive and specific estrogen genes as markers for early EC diagnosis. Our results provide promising targets for early diagnosis and treatment of EC.

## Abbreviations

EC: Endometrial cancer
EEC: endometrioid endometrial cancer
ST: spatial transcriptome
ER: estrogen receptor
DEGs: differentially expressed genes
AUC: area under ROC curve
GEO: Gene Expression Omnibus
OS: overall survival
ROC: receiver operating characteristic analysis
TCGA: The Cancer Genome Atlas
TME: tumor microenvironment
XBP1: X-box binding protein 1
AQP3: aquaporin-3
OVOL2: OVO-like zinc finger 2
CISH: cytokine inducible SH2-containing protein
STC2: Stanniocalcin-2
MREG: Melanoregulin
AREG: Amphiregulin
LR: logistic regression
GaussianNB: Gaussian naive Bayes
KNN: k-nearest neighbor
SVM: support vector machine
XGB: extreme gradient boosting
NK: neural network
AUC: receiver-operating characteristics with areas under the curve
